# Draft genome of the oriental garden lizard (*Calotes versicolor*)

**DOI:** 10.3389/fgene.2023.1091544

**Published:** 2023-02-20

**Authors:** Qing Wang, Fengping He, Ru-Yi Huang, Xueke Yang, Diancheng Yang, Jacob Njaramba Ngatia, Yanan Gong, Yanchun Xu, Song Huang, Hui Liu

**Affiliations:** ^1^ Key Laboratory of Genetics and Germplasm Innovation of Tropical Special Forest Trees and Ornamental Plants (Ministry of Education), College of Forestry, Hainan University, Haikou, China; ^2^ College of Life Sciences, University of Chinese Academy of Sciences, Beijing, China; ^3^ College of Veterinary Medicine, Yunnan Agricultural University, Kunming, China; ^4^ Shanghai Collaborative Innovation for Aquatic Animal Genetics and Breeding, Shanghai Ocean University, Shanghai, China; ^5^ Anhui Province Key Laboratory of the Conservation and Exploitation of Biological Resource, College of Life Sciences, Anhui Normal University, Wuhu, China; ^6^ College of Wildlife and Protected Area, Northeast Forestry University, Harbin, China; ^7^ Taita Hills Wildlife Sanctuary, Taita-Taveta, Kenya

**Keywords:** *Calotes versicolor*, oxford nanopore technologies (ONT), draft genome, phylogenomic analysis, reptile

## 1 Introduction

The oriental garden lizard *Calotes versicolor* (Daudin, 1802) is a medium to large-sized lizard characterized by the posterodorsal orientation of lateral scales in genus *Calotes*, subfamily Draconinae and family Agamidae (http://www.reptile-database.org/). *Calotes* is the most widely geographically distributed genus with more than 27 species being found in southern Iran, Afghanistan, Nepal, India, Sri Lanka, Brunei, Indonesia, the Malay Peninsula, Sumatra and southern China (Boulenger and Robinson, 1912; Smith, 1943; Erdelen, 1986; Zhao, 1993). Moreover, the oriental garden lizard has been introduced into Kenya, Borneo, Sulawesi, Seychelles, Mauritius, Oman and Florida (United States) (Gowande et al., 2016). The oriental garden lizard is a diurnal lizard, semi-arboreal to arboreal in habit and has been recorded mostly in scrub, deciduous forests and plantations. They usually bask in the Sun on tree trunks or branches with their heads up or down to regulate their body temperature (Diong et al., 1994). Male and female are identical in general morphology and scalation. However, females have much smaller nuchal and dorsal spines compared to the males and lack a gular sac (Pal et al., 2018). During breeding, adult males have bright body colors which can be quickly changed, and it is also called ‘chameleon tree lizard’ (Shanbhag, 2003).

*Calotes versicolor* is known to play a role in controlling insect populations, and also serves as a prey item of snakes and birds (Matyot, 2004; Sudasinghe and Somaweera, 2015). Studies on the mitochondrial DNA of *C. versicolor* conducted in Hainan island and adjacent mainland China found high genetic variation between two major lineages (Huang et al., 2013). Many researchers have mentioned the high level of morphological variation in this species across different populations, and some have considered it to be a species complex (Zug et al., 2006; Gowande et al., 2016). Recent studies have mainly focused on taxology, morphology, physiology and ecology of the *C. versicolor* (Gowande et al., 2016; Gowande et al., 2021; Tantrawatpan et al., 2021). However, the genomic background of *C*. *versicolor* is poorly characterized.

Rapid development of high-throughput sequencing technologies during the last decade has opened new avenues to address the genetic basis of adaptation and speciation in natural populations (Vijay et al., 2016). The use of genetic data has proven to be valuable in delimiting taxa that would not have been recognized based on morphology alone (Spinks and Shaffer, 2005; Wenner et al., 2012; Rodríguez et al., 2020). A high-quality genome is a valuable genetic resource to explore the possible genetic basis for the biological features of lizards (Alföldi et al., 2011; Lind et al., 2019; Gemmell et al., 2020). Therefore, obtaining the high-quality *C*. *versicolor* genome will be important for elucidating the genetic mechanisms underlying the species’ distinct biological characteristics and complexity.

Here, we generated the first high-quality genome of the oriental garden lizard using the oxford nanopore technologies (ONT) and DNBSEQ sequencing technology. This is the first and only high-quality genome of subfamily Draconinae. This high-quality *C*. *versicolor* genome with high contiguity and completeness become the genomic basis for the molecular studies in the subfamily Draconinae. It could be a valuable resource to conduct future research on the ecology, evolution and genetic mechanisms of biological characteristics of *C. versicolor* and the subfamily Draconinae.

## 2 Data briefs

In total, we generated ∼103.75 gigabases (Gb) of ONT long reads (×63 depth) for genome assembly. The average length of long reads was 15,716 bp, and the N50 of long reads was 27,282 bp. We also generated ∼222.61 Gb of clean whole genome sequencing (WGS) data (×138 depth) for genome assembly and ∼30.56 Gb of RNA-seq data for gene annotation (Table 1). Based on the prediction, the total number of 17-mer present in this subset was 186,766,705,020 and the peak depth was ×110 (Supplementary Figure S1). The *C. versicolor* genome is estimated to be 1.70 Gb in size. We yielded a draft genome assembly with 104 contigs, a total length of 1.61 Gb, and an N50 contig size of 91.60 Mb (Supplementary Table S1). Benchmarking universal Single-Copy Ortholog (BUSCO) analysis showed that 98% of 3354 BUSCO genes (vertebrata_odb10) were identified, with 97.2% single and 0.8% duplicated copy. The remaining 0.7% and 1.3% were fragmented and missing, respectively (Supplementary Table S2). This was significantly improved when compared with the published assemblies (Wilson et al., 2019) (ASM2071127v1, genome size: 0.91 Gb, contig N50: 1.62 kb and BUSCO: 6.8%, Supplementary Table S1). The *C. versicolor* genome (43.79% GC content) has a much more homogenous GC distribution in 500 kb windows than either the green anole lizard (*Anolis carolinensis*), human (*Homo sapiens*) or chicken (Gallus *Gallus*) genome (Supplementary Figure S2).

**TABLE 1:** Summary of genome assemblies and gene annotations of *Calotes versicolor* draft genome.

Item	Category	Number
**Sequencing data**	ONT (Gb)	103.75
	WGS (Gb)	222.61
	RNA-seq (Gb)	30.56
**Assembly**	Estimated genome size (Gb)	1.70
	Contigs	104
	Contig length (Gb)	1.61
	Average length (Mb)	15.52
	Minimum length (bp)	33,636
	Maximum length (Mb)	160.80
	N50 (Mb)	91.60
	GC content (%)	43.79
	BUSCO (vertebrata) complete (%)	98
**Annotation**	Repeat sequences (%)	40.29
	Number of protein-coding genes	17,547
	Number of functional annotated genes	17,546
	Average gene length (bp)	29,321.76
	Average exon length (bp)	173.85
	Average intron length (bp)	3,195.60
	Average exon per gene	9.65

In total, we identified 650.26 Mb repetitive elements representing 40.29% of our assembled *C. versicolor* genome size (Supplementary Table S3). The repeat category with the highest proportion in the genome was LTRs (22.53%) followed by LINEs (6.21%), DNA elements (6.07%), and SINEs (0.88%) (Supplementary Tables S4–S5). The final 17,547 protein-coding genes was generated by combining high-quality homology-based, *de novo*, and RNA-seq supported genes. The average gene length, exon length, and intron length were 29,321.76 bp, 173.85 bp and 3,195.60 bp, respectively (Supplementary Figure S3), which is consistent with other animals used in annotation (Table 1, Supplementary Figure S4). BUSCO analysis showed that 95.1% and 2.1% of complete and fragmented BUSCO were identified, respectively, indicative of a high-quality gene set (Supplementary Table S2). Finally, 17,546 (99.99%) protein-coding genes were functionally annotated in at least one of the five databases that were used (Supplementary Table S6). In addition, we predicted 195 miRNA, 744 tRNA, 404 rRNA and 384 snRNA in the *C. versicolor* genome, respectively (Supplementary Table S7).

**FIGURE 1:**
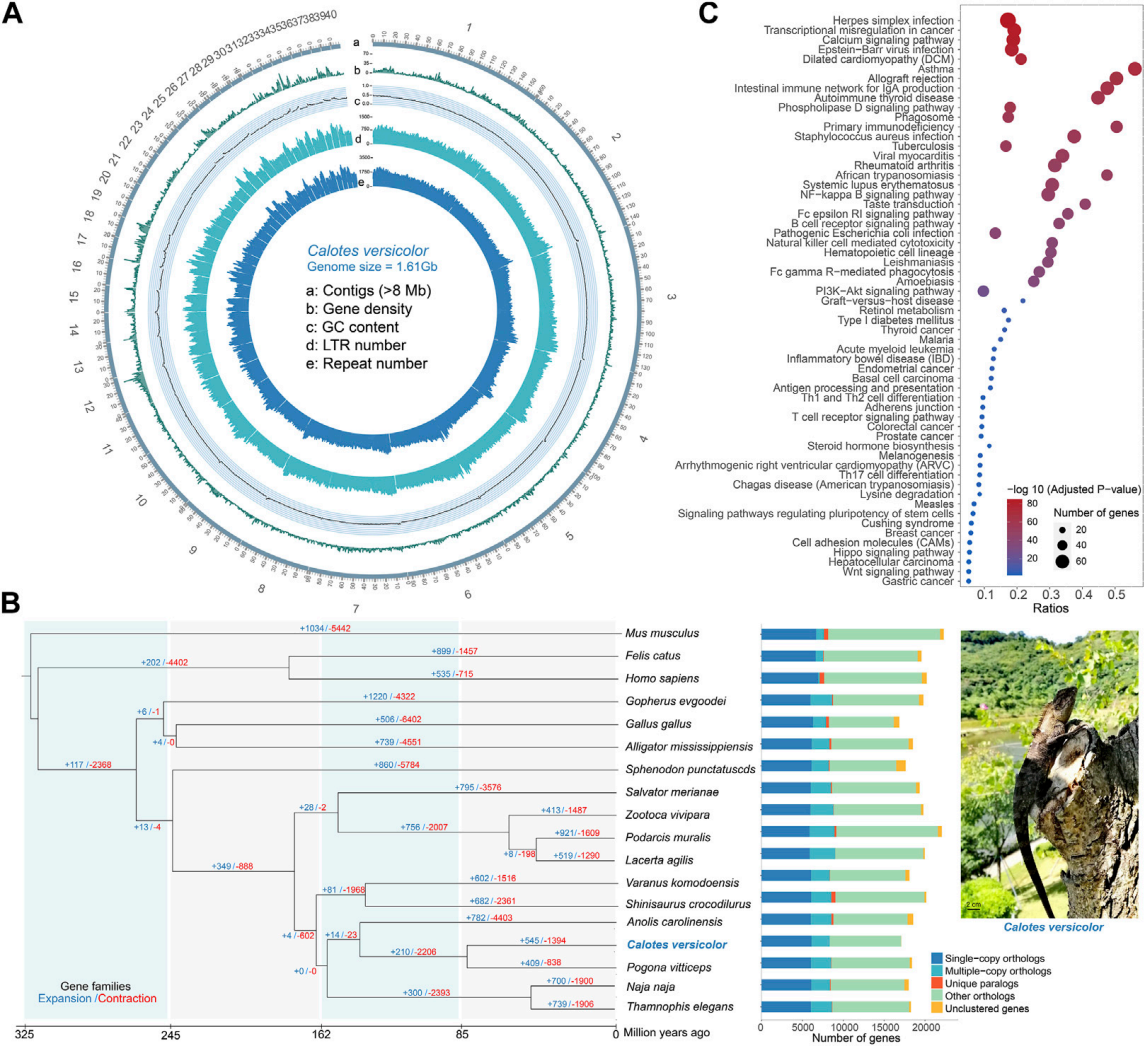
Genome landscape of the *Calotes versicolor* genome, comparative genomics analysis and enrichment analysis of expanded gene families. **(A)** Genomic features landscape of the *C. versicolor* genome (500 kb window). a: 40 contigs (92.63% of the genome) with a length range from 8.67 Mb to 160.80 Mb. b: gene count (0–61). c: GC content density (0.0011–0.6632). d: Long Terminal Repeats **(**LTR) count (0–1464). e: repeat density (2–3482). **(B)** The phylogenetic relationship of 17 species and the estimated divergence time. Gene family expansions (+) and contractions (−) are indicated by blue and red colors, respectively. The stacked bar plot presents the number of orthologous for 17 species. The right figure is adult of *C. versicolor*. **(C)** Significantly enriched 59 Kyoto Encyclopedia of Genes and Genomes (KEGG) pathways in the *C. versicolor* genome compared with the other 16 species.

Comparative genomic analyses were performed between the *C. versicolor* and 16 other species and identified 6,121 single-copy orthologs, 2,224 multiple-copy orthologs, 8,737 other orthologs and 18 unclustered genes (Figure 1B). We further identified 4,357 single-copy genes shared by these species (Supplementary Table S8). A phylogenetic tree was constructed using these genes, with divergence times being calculated between each pair of species. It was found that the *C. versicolor* and *Pogona vitticeps* is in a clade with a divergence time of 82 (35.7–122.5) million years ago (MYA), which is much later than the divergence time between the *C. versicolor* and snake (Figure 1B, Supplementary Figure S5). The gene family expansion and contraction analysis showed that 545 gene families were expanded and 1,394 gene families were contracted (Figure 1B). We performed Gene Ontology (GO) enrichment analysis of 177 genes (Supplementary Table S9) in significantly expanded gene families (*N* = 22), which showed that they were significantly enriched in 81 GO terms (Supplementary Table S10), especially those related to sensory perception and biological regulation, including sensory perception of taste (GO:0050909, *p* = 1.43E-77), chemical stimulus (GO:0007606, *p* = 1.43E-77), and sensory perception (GO:0007600, *p* = 6.93E-53). We further performed Kyoto Encyclopedia of Genes and Genomes (KEGG) enrichment analysis of these significantly expanded genes. The analysis showed that they were significantly enriched in 59 KEGG pathways (Supplementary Table S11, Figure 1C), especially those related to the immune system, including NF-kappa B signaling pathway (map04064, *p* = 1.20E-49), B cell receptor signaling pathway (map04662, *p* = 6.68E-42), Natural killer cell mediated cytotoxicity (map04650, *p* = 2.29E-40) and T cell receptor signaling pathway (map04660, *p* = 1.14E-4). The biological characteristics, included Taste transduction (map04742, *p* = 3.06E-46), Retinol metabolism (map00830, *p* = 4.36E-08) and Melanogenesis (map04916, *p* = 1.87E-4).

## 3 Materials and methods

### 3.1 Sample collection and sequencing

An adult male oriental garden lizard was collected from car crash in Haikou, Hainan Province, China. The muscle sample was collected, transferred to liquid nitrogen immediately and stored at −80°C for long fragment DNA isolation. Procedures were approved by the College of Forestry, Hainan University (HNUAUCC-2022–000151). For the Nanopore library, 8–10 µg of genomic DNA was purified (>50 kb) with the SageHLS HMW library system (Sage Science). DNA libraries (∼800 ng) were constructed and sequenced on the PromethION (Oxford Nanopore Technologies, ONT) at the NOVOGENE (Beijing, China). Additionally, total RNA from a pooling sample of three different tissues (blood, muscle and skin) was extracted using TRlzol reagent (Invitrogen, United States) according to the manufacturer’s instructions. RNA integrity and purity was measured by a Qubit 3.0 Fluorometer (Life Technologies, United States). cDNA libraries were reverse transcribed from 200 to 400 bp RNA fragments. For whole genome sequence (WGS) library, total genomic DNA was extracted using a DNeasy Blood and Tissue Kit (Qiagen, United States). Both RNA ang WGS were subjected to paired-end sequencing using a DNBSEQ-T1 sequencer (MGI tech, Shenzhen, Guangdong, China).

### 3.2 K-mer distribution and genome size estimation

To estimate the genome size of the *C. versicolor*, we generated the k-mer depth distribution with a 17 bp k-mer size using DNBSEQ short reads (∼222.61 Gb) were analyzed by KmerFreq v1.0, (Liu et al., 2013). The K-mer frequency distribution was plotted in R v3.5.1, ‘ggplot2’ package (Villanueva and Chen, 2019). The genome size of *C. versicolor* was estimated using the following formula: estimated genome size = kmer_num/pkdepth, where the kmer_num is the total number of k-mers, and pkdepth refers to the most frequent peak. According to that prediction, the total number of 17-mer present in this subset was 186,766,705,020 and the peak depth was ×110. The size of the *C. versicolor* genome was estimated to be 1.70 Gb.

### 3.3 Genome assembly

Assembly was performed using the NextDenovo v 2.5.0, (https://github.com/Nextomics/NextDenovo). Meanwhile, NextDenovo contains two core modules: NextCorrect and NextGraph. The NextCorrect module was used for raw Nanopore long-reads correction and consensus sequence extraction. The NextGraph module was used for preliminary assembly (Hu et al., 2020). The read cutoff was set at 1 kb for the NextDenovo genome assembly, while default parameters were used for other settings. The primary assembly further improved the single base accuracy using NextPolish v1.4.0, (Hu et al., 2020), with all parameters set to default. At the genome polishing stage, Nanopore long-reads were used repetitively two times, and DNBSEQ short reads were used four times for genome correction. The completeness of the genome and gene set were evaluated by Benchmarking universal Single-Copy Orthologs (BUSCO, v3.1.0) (Simão et al., 2015) analysis using the database of vertebrata_odb10. GC content was measured in non-overlapping 500 kb windows in *C. versicolor*, *A. carolinensis*, *H. sapiens* and *G. Gallus* genome. In the window, GC% was (G + C)/(A + C + G + T).

### 3.4 Repeat regions prediction and classification

Refrom each species was used to performpetitive elements were identified using a combination of homology-based and *de novo* approaches. For the homology-based approach at both the DNA and protein levels, the genome assembly was aligned to the known repeat database RepBbase using RepeatMasker v4.0.5, (Chen, 2004), RepeatProteinMask (Chen, 2004) and Tandem Repeats Finder v4.07b, (Ou and Jiang, 2018). For the *de novo*-based approach, RepeatModeler v2.0, (Flynn et al., 2020), and LTR_retriever (Ou and Jiang, 2018) were used to construct a *de novo* repeat library. All repetitive elements were masked for gene annotation. We also mapped the gene density, GC content, LTR density and repeat density onto 40 contigs (length >8.67 Mb and 92.63% of the *C. versicolor* genome) using the CIRCOS v0.69–8, (Krzywinski et al., 2009).

### 3.5 Protein-coding gene prediction and annotation

We predict assembled gene set combined homology-based, *de novo* and transcriptome-based methods. In the homology-based method, protein sequences of *G. Gallus*, *H. sapiens*, *A. carolinensis*, *P. vitticeps*, *Varanus komodoensis* and *Podarcis muralis* available in the NCBI database were mapped to the *C. versicolor* genome using GeneWise v2.4.1, (Birney et al., 2004), with an E-value cutoff of 1e-5. In the *de novo* method, we ran the repeat-masked genome using Augustus v3.0.3, (Stanke et al., 2004). In the transcriptome-based method, transcripts were assembled using StringTie v1.3.3b, (Pertea et al., 2015), based on clean RNA-seq data. The final protein-coding gene set was generated using the MAKER pipeline v3.01.03, (Campbell et al., 2014), by combining high-quality homology-based, *de novo* and RNA-seq supported genes. The completeness of the genome and gene set were evaluated through BUSCO analysis using the database of vertebrata_odb10. Functional annotations of protein-coding genes were carried out using BLAST (e-value cut-off of 1e-5) against publicly available databases including the Swiss-Prot, TrEMBL and KEGG database. InterProScan v5.52–86.0, (Jones et al., 2014), was used to predict motifs and domains, as well as Gene ontology (GO) terms. In addition, non-coding RNA genes, including tRNA, miRNA, snRNA and rRNA, were predicted in the assembled genome. tRNA genes were identified using tRNAscan-SE v1.3.1, (Lowe and Eddy, 1997). SnRNA and miRNA genes were detected by searching the reference genome sequences against the content of the Rfam database (Release 12.0) using BLAST. The rRNA genes were detected by alignment with BLASTN against known human rRNA sequences, with an e-value of 1e-5.

### 3.6 Phylogenetic and gene family analysis

We performed a comparative genomic analysis between the *C. versicolor* and 16 other species with an outgroup *Mus musculus* (Supplementary Table S8). First, the longest transcript of each gene from each species was used to perform all-to-all BLAST v2.2.26, (Mount, 2007), analysis with the parameter “-p blastp -m8 -e 1e-5 -F F”. Then, genes were clustered using Treefam v1.4, (Li et al., 2006), pipeline with hierarchically clustering on a sparse graph. Finally, 21,771 gene families were identified in all 17 reference genomes, with 4,357 single-copy genes being shared by these 17 species. These single-copy genes were used to construct a Maximum-Likelihood (ML) phylogenetic tree using IQTREE v1.6.12, (Nguyen et al., 2015), with the best-fit evolutionary substitution model (GTR + F + R4) that was evaluated using ModelFinder (Kalyaanamoorthy et al., 2017). To estimate the divergence time between *C. versicolor* and other 16 species, we used MCMC Tree v4.5, (Yang, 2007), implemented in the PAML package. Sequences for 4,357 single-copy genes were used as the input file for MCMC Tree, and multiple fossil times (*G. gallus*-*H. sapiens*: 312.3–330.4, *G. gallus-A. carolinensis*: 259.7–299.8, *G. gallus*-*P. vitticeps*: 276.0–286.8, *G. gallus*-*Alligator mississippiensis*: 240.9–247.0, *Naja naja*-*A. carolinensis*: 148.5–166.4 and *A. carolinensis*-*P. vitticeps*:135.0–160.5) were used for time calibrations from Timetree (http://www.timetree.org/). The Markov chain Monte Carlo (MCMC) process was run for 1,500,000 iterations with a sampling frequency of 150 after aburn-in of 500,000 iterations.

Using the divergence time ranges between the 17 species and the inferred phylogenetic tree, the expanded and contracted gene families were detected using CAFÉ v4.2.1, (De Bie et al., 2006). KEGG enrichment analyses were performed on the expanded gene families with all annotated genes used as the background. Fisher’s exact test was used to improve the accuracy of the conducted χ^2^ tests. Finally, the Benjamini–Hochberg method (Peng et al., 2017) was used to generate adjusted *p*-values.

## Data Availability

The datasets presented in this study can be found in online repositories. The names of the repository/repositories and accession number(s) can be found below: China National GeneBank DataBase Sequence Archive (https://db.cngb.org/cnsa/) of the China National GeneBank DataBase under accession number CNP0003598.

## References

[B1] Alföldi J. Di Palma F. Grabherr M. Williams C. Kong L. Mauceli E. (2011). The genome of the green anole lizard and a comparative analysis with birds and mammals. Nature 477 (7366), 587–591. 10.1038/nature10390 21881562 PMC3184186

[B2] Birney E. Clamp M. Durbin R. (2004). GeneWise and genomewise. Genome Res. 14 (5), 988–995. 10.1101/gr.1865504 15123596 PMC479130

[B3] Boulenger G.-A. Robinson H. C. (1912). A vertebrate fauna of the Malay Peninsula from the Isthmus of Kra to Singapore including the adjacent islands. Nature 90, 619. 10.1038/090619a0

[B4] Campbell M. S. Holt C. Moore B. Yandell M. (2014). Genome annotation and curation using MAKER and MAKER‐P. Curr. Protoc. Bioinforma. 48 (1), 4.11.111–4.11.3914.11. 39. 10.1002/0471250953.bi0411s48 PMC4286374 25501943

[B5] Chen F. Z. You L. J. Yang F. Wang L. N. Guo X. Q. Gao F. (2020). CNGBdb: China national genebank database. Yi Chuan= Hered. 42 (8), 799–809. 10.16288/j.yczz.20-080 32952115

[B6] Chen N. (2004). Using Repeat Masker to identify repetitive elements in genomic sequences. Curr. Protoc. Bioinforma. 5 (1), 4–14.10. 14. 10.1002/0471250953.bi0410s05 18428725

[B7] De Bie T. Cristianini N. Demuth J. P. Hahn M. W. (2006). Cafe: A computational tool for the study of gene family evolution. Bioinformatics 22 (10), 1269–1271. 10.1093/bioinformatics/btl097 16543274

[B8] Diong C. Chou L. Lim K. (1994). *Calotes versicolor*: The changeable lizard. Malaysia): Nature Malaysia.

[B9] Erdelen W. (1986). The genus *Calotes* (Sauria: Agamidae) in Sri Lanka: Clutch sizes and reproductive seasonality of *Calotes versicolor*-preliminary results. Spixiana 9 (1), 111–115.

[B10] Flynn J. M. Hubley R. Goubert C. Rosen J. Clark A. G. Feschotte C. (2020). RepeatModeler2 for automated genomic discovery of transposable element families. Proc. Natl. Acad. Sci. 117 (17), 9451–9457. 10.1073/pnas.1921046117 32300014 PMC7196820

[B11] Gemmell N. J. Rutherford K. Prost S. Tollis M. Winter D. Macey J. R. (2020). The tuatara genome reveals ancient features of amniote evolution. Nature 584 (7821), 403–409. 10.1038/s41586-020-2561-9 32760000 PMC7116210

[B12] Gowande G. Mishra A. Mirza Z. A. (2016). Neotype designation for *Calotes versicolor* daudin, 1802 (sauria: Agamidae) with notes on its systematics. Zootaxa 4126 (2), 271–279. 10.11646/zootaxa.4126.2.7 27395587

[B13] Gowande G. Pal S. Jablonski D. Masroor R. Phansalkar P. U. Dsouza P. (2021). Molecular phylogenetics and taxonomic reassessment of the widespread agamid lizard *Calotes versicolor* (Daudin, 1802)(Squamata, Agamidae) across South Asia. Vertebr. Zool. 71, 669–696. 10.3897/vz.71.e62787

[B14] Guo X. Chen F. Gao F. Li L. Liu K. You L. (2020). Cnsa: A data repository for archiving omics data. Database 2020, baaa055. 10.1093/database/baaa055 32705130 PMC7377928

[B15] Hu J. Fan J. Sun Z. Liu S. (2020). NextPolish: A fast and efficient genome polishing tool for long-read assembly. Bioinformatics 36, 2253–2255. 10.1093/bioinformatics/btz891 31778144

[B16] Huang Y. Guo X. Ho S. Y. Shi H. Li J. Li J. (2013). Diversification and demography of the oriental garden lizard (*Calotes versicolor*) on Hainan Island and the adjacent mainland. PLoS One 8 (6), e64754. 10.1371/journal.pone.0064754 23840304 PMC3694074

[B17] Jones P. Binns D. Chang H. Y. Fraser M. Li W. McAnulla C. (2014). InterProScan 5: Genome-scale protein function classification. Bioinformatics 30 (9), 1236–1240. 10.1093/bioinformatics/btu031 24451626 PMC3998142

[B18] Kalyaanamoorthy S. Minh B. Q. Wong T. K. Von Haeseler A. Jermiin L. S. (2017). ModelFinder: Fast model selection for accurate phylogenetic estimates. Nat. methods 14 (6), 587–589. 10.1038/nmeth.4285 28481363 PMC5453245

[B19] Krzywinski M. Schein J. Birol I. Connors J. Gascoyne R. Horsman D. (2009). Circos: An information aesthetic for comparative genomics. Genome Res. 19 (9), 1639–1645. 10.1101/gr.092759.109 19541911 PMC2752132

[B20] Li H. Coghlan A. Ruan J. Coin L. J. Heriche J. K. Osmotherly L. (2006). TreeFam: A curated database of phylogenetic trees of animal gene families. Nucleic acids Res. 34, D572–D580. 10.1093/nar/gkj118 16381935 PMC1347480

[B21] Lind A. L. Lai Y. Y. Mostovoy Y. Holloway A. K. Iannucci A. Mak A. C. (2019). Genome of the Komodo dragon reveals adaptations in the cardiovascular and chemosensory systems of monitor lizards. Nat. Ecol. Evol. 3 (8), 1241–1252. 10.1038/s41559-019-0945-8 31358948 PMC6668926

[B22] Liu B. Shi Y. Yuan J. Hu X. Zhang H. Li N. (2013). Estimation of genomic characteristics by analyzing k-mer frequency in de novo genome projects. *arXiv preprint arXiv:1308.2012* .

[B23] Lowe T. M. Eddy S. R. (1997). tRNAscan-SE: a program for improved detection of transfer RNA genes in genomic sequence. Nucleic acids Res. 25 (5), 955–964. 10.1093/nar/25.5.955 9023104 PMC146525

[B24] Matyot P. (2004). The establishment of the crested tree lizard, *Calotes versicolor* (Daudin, 1802)(Squamata: Agamidae) in Seychelles. Phelsuma 12, 35–47.

[B25] Mount D. W. (2007). Using the basic local alignment search tool (BLAST). Cold Spring Harb. Protoc. 2007 (7), top17. 10.1101/pdb.top17 21357135

[B26] Nguyen L. T. Schmidt H. A. Von Haeseler A. Minh B. Q. (2015). IQ-TREE: A fast and effective stochastic algorithm for estimating maximum-likelihood phylogenies. Mol. Biol. Evol. 32 (1), 268–274. 10.1093/molbev/msu300 25371430 PMC4271533

[B27] Ou S. Jiang N. (2018). LTR_retriever: A highly accurate and sensitive program for identification of long terminal repeat retrotransposons. Plant physiol. 176 (2), 1410–1422. 10.1104/pp.17.01310 29233850 PMC5813529

[B28] Pal S. Vijayakumar S. Shanker K. Jayarajan A. Deepak V. (2018). A systematic revision of *Calotes Cuvier*, 1817 (Squamata: Agamidae) from the Western Ghats adds two genera and reveals two new species. Zootaxa 4482 (3), 401–450. 10.11646/zootaxa.4482.3.1 30313808

[B29] Peng J. Liu W. Bretz F. Shkedy Z. (2017). Multiple confidence intervals for selected parameters adjusted for the false coverage rate in monotone dose-response microarray experiments. Biometrical J. 59 (4), 732–745. 10.1002/bimj.201500254 28025852

[B30] Pertea M. Pertea G. M. Antonescu C. M. Chang T. C. Mendell J. T. Salzberg S. L. (2015). StringTie enables improved reconstruction of a transcriptome from RNA-seq reads. Nat. Biotechnol. 33 (3), 290–295. 10.1038/nbt.3122 25690850 PMC4643835

[B31] Rodríguez A. Rodríguez B. Montelongo T. Garcia‐Porta J. Pipa T. Carty M. (2020). Cryptic differentiation in the Manx shearwater hinders the identification of a new endemic subspecies. J. Avian Biol. 51 (11), jav.02633. 10.1111/jav.02633

[B32] Shanbhag B. A. (2003). Reproductive strategies in the lizard, *Calotes versicolor* . Curr. Sci. 2003, 646–652.

[B33] Simão F. A. Waterhouse R. M. Ioannidis P. Kriventseva E. V. Zdobnov E. M. (2015). BUSCO: Assessing genome assembly and annotation completeness with single-copy orthologs. Bioinformatics 31 (19), 3210–3212. 10.1093/bioinformatics/btv351 26059717

[B34] Smith M. A. (1943). The fauna of British India including Ceylon and Burma. Red Lion Court, Fleet St.; London: Taylor and Francis.

[B35] Spinks P. Q. Shaffer H. B. (2005). Range‐wide molecular analysis of the Western pond turtle (Emys marmorata): Cryptic variation, isolation by distance, and their conservation implications. Mol. Ecol. 14 (7), 2047–2064. 10.1111/j.1365-294X.2005.02564.x 15910326

[B36] Stanke M. Steinkamp R. Waack S. Morgenstern B. (2004). Augustus: A web server for gene finding in eukaryotes. Nucleic acids Res. 32, W309–W312. 10.1093/nar/gkh379 15215400 PMC441517

[B37] Sudasinghe H. Somaweera R. (2015). *Calotes versicolor* (oriental garden lizard). Diet. Herpetol. Rev. 46, 625–629.

[B38] Tantrawatpan C. Thongnetr W. Pilap W. Suksavate W. Agatsuma T. Tawong W. (2021). Genetic diversity and population structure of the oriental garden lizard, *Calotes versicolor* Daudin, 1802 (Squamata: Agamidae) along the Mekong River in Thailand and Lao PDR. Asian Herpetological Res. 12 (1), 49–57.

[B39] Vijay N. Bossu C. M. Poelstra J. W. Weissensteiner M. H. Suh A. Kryukov A. P. (2016). Evolution of heterogeneous genome differentiation across multiple contact zones in a crow species complex. Nat. Commun. 7 (1), 13195. 10.1038/ncomms13195 27796282 PMC5095515

[B40] Villanueva R. A. M. Chen Z. J. (2019). ggplot2: elegant graphics for data analysis. Measurement: Interdisciplinary Research and Perspectives 17 (3), 160–167. 10.1080/15366367.2019.1565254

[B41] Wenner T. J. Russello M. A. Wright T. F. (2012). Cryptic species in a neotropical parrot: Genetic variation within the Amazona farinosa species complex and its conservation implications. Conserv. Genet. 13 (5), 1427–1432. 10.1007/s10592-012-0364-8

[B42] Wilson C. A. Titus T. Batzel P. Postlethwait J. H. Raman R. (2019). A search for sex-linked loci in the agamid lizard, *Calotes versicolor* . Sex. Dev. 13 (3), 143–150. 10.1159/000500465 31247625

[B43] Yang Z. (2007). Paml 4: Phylogenetic analysis by maximum likelihood. Mol. Biol. Evol. 24 (8), 1586–1591. 10.1093/molbev/msm088 17483113

[B44] Zhao E. M. (1993). Herpetology of China. Contrib. Herpetol. 10, 1–522.

[B45] Zug G. R. Brown H. H. Schulte J. A. Vindum J. V. (2006). Systematics of the garden lizards, *Calotes versicolor* group (Reptilia, Squamata, Agamidae), in Myanmar: Central dry zone populations. Proc. Calif. Acad. Sci. 57, 35–68.

