# Supplementary material for: Draft genome of the oriental garden lizard (Calotes versicolor)
Source: Front Genet. 2023 Feb 20;14:1091544. doi: 10.3389/fgene.2023.1091544 (PMC9986473; doi:10.3389/fgene.2023.1091544)
Supplement: Supplementary file 1 [file DataSheet1.pdf]

Supplementary Material

1 Supplementary Figures and Tables

1.1 Supplementary Figures

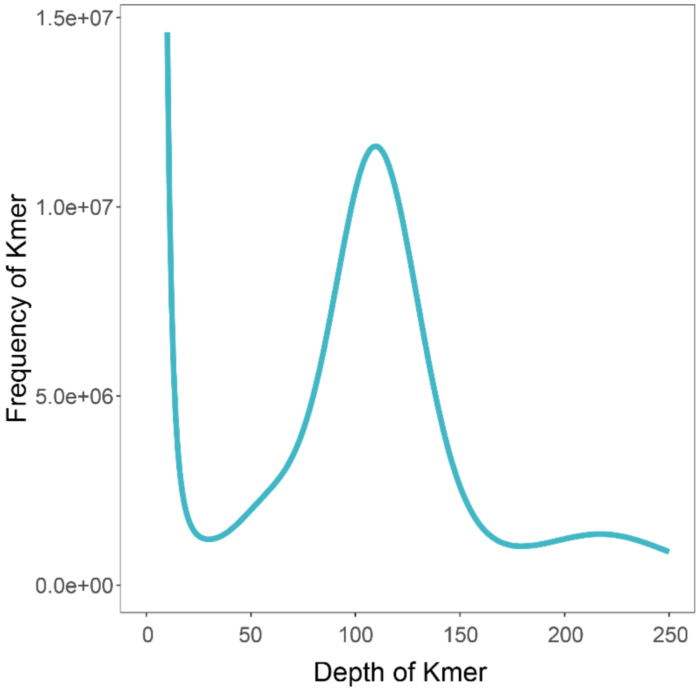

Supplementary Figure 1. K-mer frequency distribution at k-mer size of 17. K-mer refers to an artificial sequence division of K nucleotides. The peak depth was 110X. The total number of 17-mer present in this subset was 186,766,705,020. The genome size was estimated to be 1.70 Gb according to the following formula: genome size = (Kmer number)/(Peak depth).

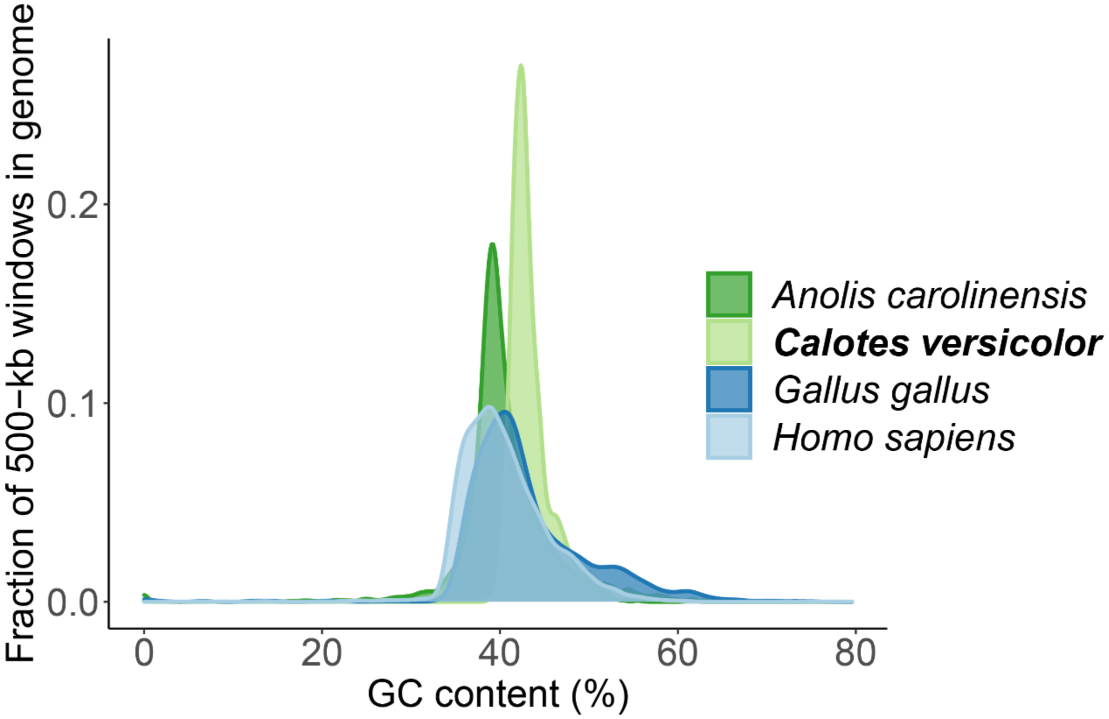

Supplementary Figure 2. *C. versicolor*, *Anolis carolinensis*, *Homo sapiens* and *Gallus gallus* genomes display very different GC distributions. Each line depicts the distribution of GC content of the *C. versicolor*, *Anolis carolinensis*, *Homo sapiens* or *Gallus gallus* genome when divided into 500 kb windows.

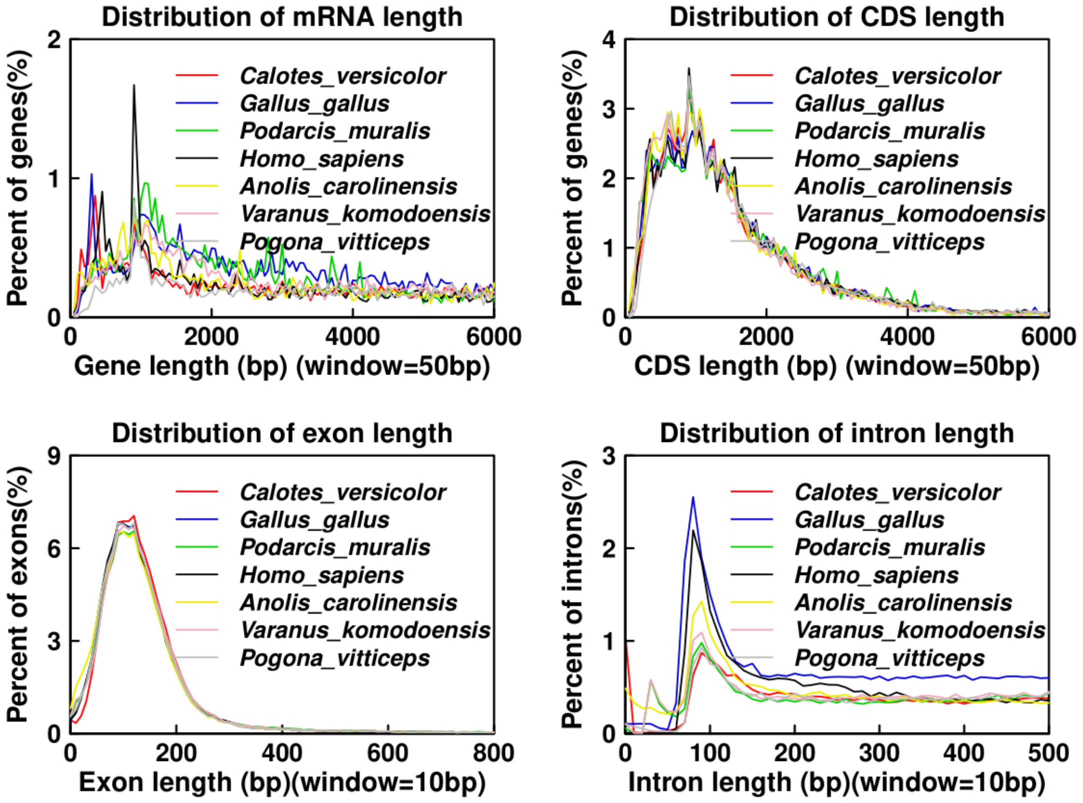

Supplementary Figure 3. Comparisons of CDS length, mRNA length, intron length and exon length among 7 species.

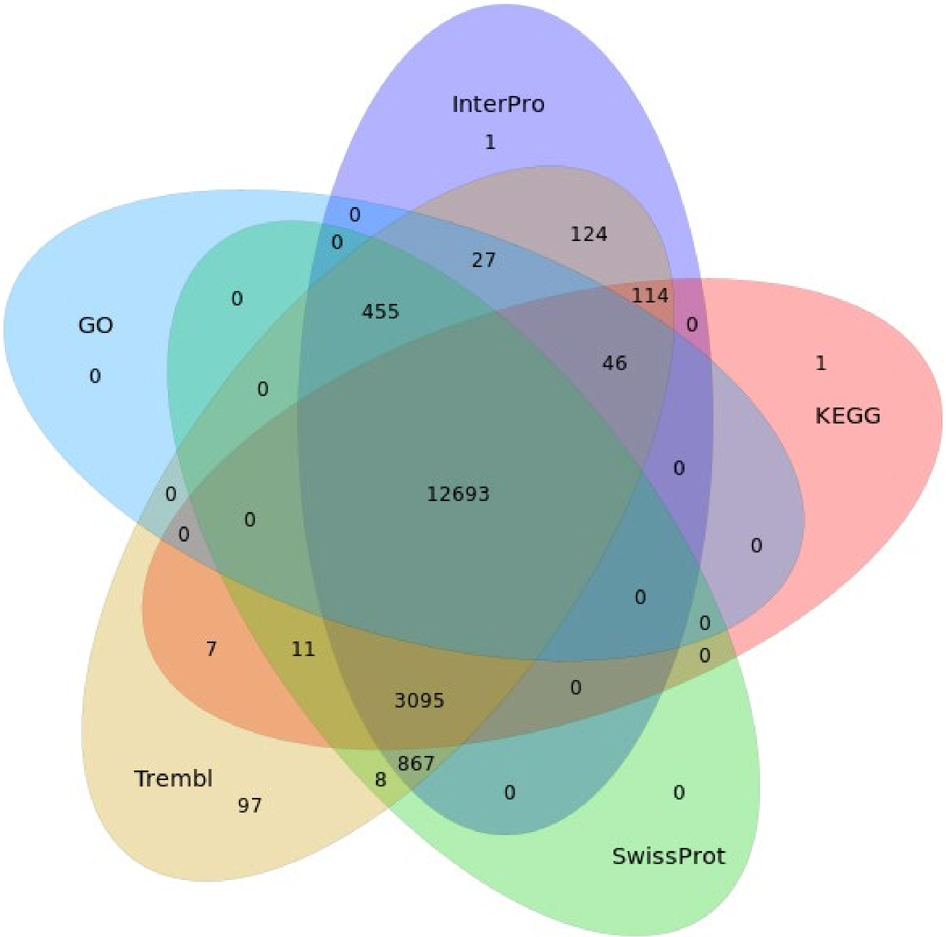

Supplementary Figure 4. Venn diagram representing the functional annotation of the *C. versicolor* gene set.

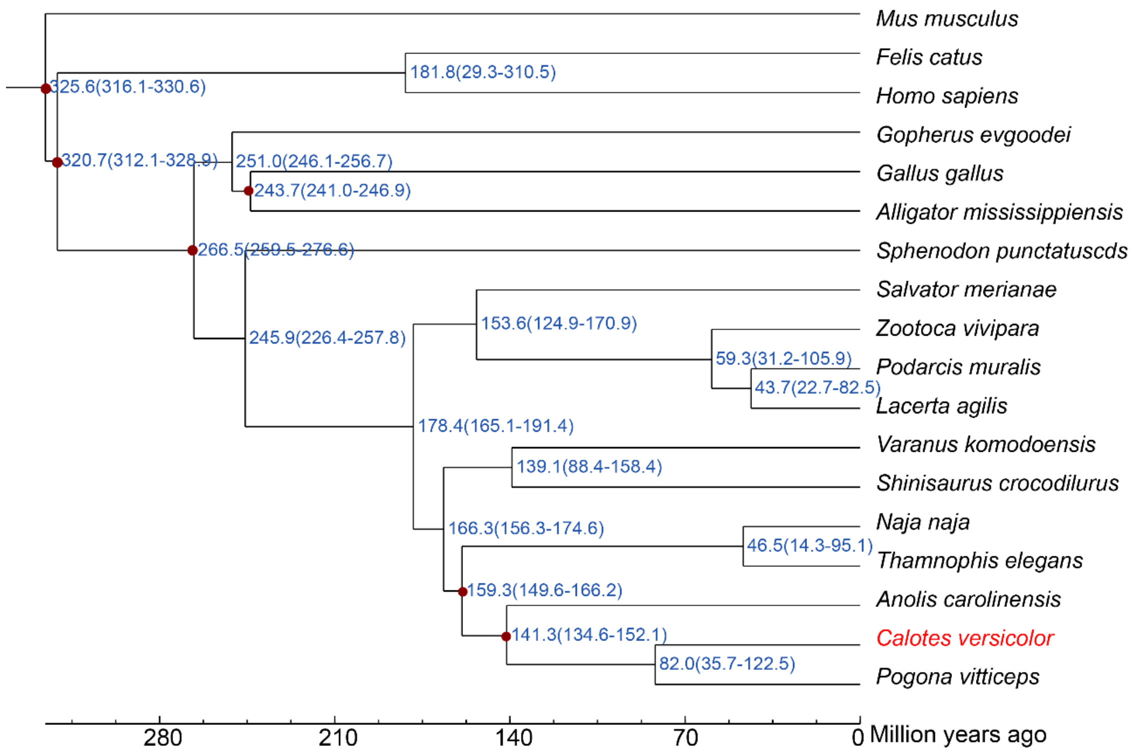

Supplementary Figure 5. Phylogenetic tree of the *C. versicolor* with divergent time. The numerical interval at the nodes represent 95% confidence intervals of the corresponding estimated divergence times. Red circles at each node represent calibration points listed in Method.

1.2 Supplementary Tables

Supplementary Table 1. Comparison of assembly statistics among our assembled *C. versicolor* genome, the previously published *C. versicolor* genome.

Assembly Level	Parameters	Current assembly	ASM2071127v1
Contig	Maximal length (bp)	160,793,834	34,655
	N75 (bp)	8,981,137	1,015
	N50 (bp)	91,599,063	1,622
	number	104	667,617
	number>=1kp	104	345,158
	number>=50kb	101	0
	Genome size (Gb)	1.614	0.913
	BUSCO (vertebrata_odb10)	98.0%	6.8%

Supplementary Table 2. Result of BUSCO analysis of the *C. versicolor* genome.

Level	BUSCO assessment results	Dataset	Parameter
Genome	C:98.0%[S:97.2%,D:0.8%],F:0.7%,M:1.3%,n:3354	vertebrata_odb10	-m genome
Gene	C:95.1%[S:93.8%,D:1.3%],F:2.1%,M:2.8%,n:3354	vertebrata_odb10	-m proteins

Supplementary Table 3. Statistics of Repeats in the *C. versicolor* genome.

**Type**	**Length (bp)**	**% of genome**
Trf	73,311,685	4.54
Repeatmasker	243,443,011	15.08
Proteinmask	123,756,776	7.67
De novo	581,714,530	36.04
Total	650,258,091	40.29

Supplementary Table 4. Statistics of identified Repeats by *De novo* method in *C. versicolor* Genome.

**Type**	**Length (bp)**	**% of genome**
DNA	98,023,377	6.07
LINE	100,186,040	6.21
SINE	14,281,452	0.88
LTR	363,603,862	22.53
Other	0	0.00
Satellite	0	0.00
Simple_repeat	583,362	0.04
Unknown	51,192,498	3.17
Total	581,714,530	36.04

Supplementary Table 5. Transposable elements (TEs) in the *C. versicolor* genome assembly.

	**Repbase TEs**		**TE proteins**		***De novo***		**Combined TEs**	
**Type**	Length (bp)	% in genome	Length (bp)	% in genome	Length (bp)	% in genome	Length (bp)	% in genome
**DNA**	76,198,136	4.72	226,521	0.01	98,023,377	6.07	157,291,284	9.74

**LINE**	140,062,583	8.68	105,796,03[illegible]	6.55	100,186,04[illegible]	6.21	185,971,65[illegible]	11.52
**SINE**	15,402,404	0.95	0	0.00	14,281,452	0.88	20,865,488	1.29
**LTR**	25,471,680	1.58	17,761,134	1.10	363,603,86[illegible]	22.53	366,430,61[illegible]	22.70
**Other**	35,188	0.00	0	0.00	0	0.00	35,188	0.00
**Unknown**	0	0.00	0	0.00	51,192,498	3.17	51,192,498	3.17
**Total**	243,443,011	15.08	123,756,77[illegible]	7.67	581,131,16[illegible]	36.00	628,791,14[illegible]	38.96

Note: Repbase TEs: the result of RepeatMasker based on Repbase; TE proteins: the result of RepeatProteinMask based on Repbase; *De novo*: *de novo* finding repeats (Repeatmodeler and ltr_retriever); Combined TEs: the results obtained from combining the results using all the approaches.

Supplementary Table 6. Statistics on functional annotation of the *C. versicolor* gene set.

Values	Total	Swissprot-[illegible]	KEGG-[illegible]	TrEMBL-[illegible]	Interpro-[illegible]	GO-[illegible]	Overall
**Number**	17,547	17,129	15,967	17,544	17,422	13,221	17,546
**Percentage**	100%	97.62%	91.00%	99.98%	99.29%	75.35%	99.99%

Supplementary Table 7. Statistics of non-coding RNA annotation.

Type		number	Average length (bp)	Total length (bp)	% of genome
**miRNA**		195	83.19	16,222	0.001005
**tRNA**		744	76.66	57,036	0.003534
**rRNA**	rRNA	404	262.83	106,185	0.006579
	18S	79	385.67	30,468	0.001888
	28S	202	317.80	64,196	0.003977
	5.8S	17	152.00	2,584	0.00016
	5S	106	84.31	8,937	0.000554
**snRNA**	snRNA	384	131.87	50,639	0.003137
	CD-box	100	100.21	10,021	0.000621
	HACA-box	69	146.99	10,142	0.000628
	splicing	201	139.07	27,954	0.001732

Supplementary Table 8. Species used in comparative genomics analysis.

Common Name	Latin Name	Genebank id	Genome

American alligator	*Alligator mississippiensis*	GCF_000281125.3	ASM28112v4
Argentine black and white tegu	*Salvator merianae*	GCA_003586115.1	HLtupMer3
Bearded dragon	*Pogona vitticeps*	GCF_900067755.1	pvi1.1
Chicken	*Gallus gallus*	GCF_016699485.2	bGalGal1.mat.broiler.GRCg7b
Chinese crocodile lizard	*Shinisaurus crocodilurus*	GCA_021292165.1	IOZ_Scro_1.0
Common wall lizard	*Podarcis muralis*	GCF_004329235.1	PodMur_1.0
Domestic cat	*Felis_catus*	GCA_000181335.5	Felis_catus_9.0
Garter Snake	*Thamnophis elegans*	GCF_009769535.1	rThaEle1.pri
Goodes thornscrub tortoise	*Gopherus evgoodei*	GCA_007399415.1	rGopEvg1_v1
Green anole	*Anolis carolinensis*	GCF_000090745.1	AnoCar2.0
House mouse	*Mus musculus*	GCF_000001635.20	GRCm38
Human	*Homo sapiens*	GCA_000001405.29	GRCh38.p14
Indian cobra	*Naja naja*	GCA_009733165.1	Nana_v5
Komodo dragon	*Varanus komodoensis*	GCF_004798865.1	ASM479886v1
Oriental Garden Lizard	*Calotes versicolor*	/	**This study**
Sand Lizard	*Lacerta agilis*	GCF_009819535.1	rLacAgi1.pri
Tuatara	*Sphenodon punctatus*	GCA_003113815.1	ASM311381v1
Viviparous lizard	*Zootoca vivipara*	GCF_011800845.1	UG_Zviv_1

Supplementary Table 9. Annotated genes of contracted ($N = 39$) and expanded ($N = 117$) gene families in the *C. versicolor* genome.

Contraction	Expansion				
CP2AD	ALP1	IGHV4-30-4	LOC110091621	PARPI-0050072	ZN557
CP2C8	ALPL	IGHV4-38-2	LOC110091623	RDH16	ZN558
CP2CT	AT3G55350	IGHV4-39	LOC114583321	RDH7	ZN772
HMR1	C3	IGHV4-59	LOC114586355	SETMAR	ZN792
O1019	CGPICR-022873	IGHV6-1	LV1	SETMR	ZN844
O1020	DNAJ	IGKV3-15	LV140	T2R10	ZN862
O1086	DNAJB2	IGKV3-20	LV151	T2R39	ZNF157
O10A7	DNJB2	IGKV3D-20	LV208	T2R40	ZNF177
O10C1	DR999-PMT21178	IGL1	LV218	T2R41	ZNF20
O11A1	H17B6	IGLC6	LV223	TA2R4	ZNF316
O11G2	HSD17B6	IGLV11-55	LV301	TA2R7	ZNF426
O11L1	HV01	IGLV1-40	LV310	TA2R8	ZNF433
O14AG	HV03	IGLV1-51	LV319	TA2R9	ZNF440
O14J1	HV205	IGLV2-18	LV321	TAS2R10	ZNF534
OLF1	HV226	IGLV2-23	LV39	TAS2R104	ZNF557
OR1F1	HV307	IGLV2-8	LV469	TAS2R134	ZNF558
OR5V1	HV311	IGLV3-1	LV537	TAS2R143	ZNF77
OR9G4	HV333	IGLV3-10	LV545	TAS2R39	ZNF772
PRS27	HV348	IGLV3-19	LV552	TAS2R4	ZNF792

PRS33	HV366	IGLV3-21	LV861	TAS2R40	ZNF844
SCND3	HV439	IGLV3-9	LV949	TAS2R41	ZNF862
TIGD1	HV459	IGLV4-69	LVK55	TAS2R7	
TRI11	HV601	IGLV5-37	MDA-GLEAN10000198	TAS2R8	
TRI27	HVC33	IGLV5-45	MOS1T	TAS2R9	
TRI39	HVD34	IGLV5-52	O1019	TR104	
TRIM7	HVD82	IGLV8-61	O1020	TR134	
V2R26	HVM43	IGLV9-49	O10A7	TR143	
ZKSC7	HVM44	IGW3	O11G2	VPRE2	
ZN202	HVM45	KV315	O5AS1	VPREB2	
ZN287	HVM60	KV320	OLFR1019	XERD	
ZN420	IGHV2-26	KV5A2	OLFR1020	ZF316	
ZN436	IGHV2-5	KVD20	OR10A7	ZIK1	
ZN569	IGHV3-11	LAC1	OR11G2	ZN157	
ZN665	IGHV3-30-3	LOC100557315	OR4M1	ZN177	
ZN850	IGHV3-33	LOC100558368	OR5AS1	ZN316	
ZNF24	IGHV3-48	LOC110070527	PARPI-0018667	ZN426	
ZNF85	IGHV3-6	LOC110077393	PARPI-0029683	ZN433	
ZSC31	IGHV3-66	LOC110081179	PARPI-0030191	ZN440	

Supplementary Table 10. Results of the GO enrichment analysis of expanded gene families in the *C. versicolor* genome.

Ontology	ID	GO Term	Adjusted P value
BP	GO:0050909	sensory perception of taste	1.43E-77
BP	GO:0007606	sensory perception of chemical stimulus	1.43E-77
BP	GO:0007186	G protein-coupled receptor signaling pathway	1.15E-74
MF	GO:0004888	transmembrane signaling receptor activity	5.09E-56
BP	GO:0007600	sensory perception	6.93E-53
BP	GO:0032501	multicellular organismal process	8.53E-53
BP	GO:0050877	nervous system process	1.18E-52
MF	GO:0038023	signaling receptor activity	1.5E-51
MF	GO:0060089	molecular transducer activity	5.85E-51
BP	GO:0003008	system process	2.54E-47
MF	GO:0004930	G protein-coupled receptor activity	1.05E-34
BP	GO:0050794	regulation of cellular process	8.9E-32
BP	GO:0015074	DNA integration	3.27E-30
BP	GO:0050789	regulation of biological process	1.31E-29
BP	GO:0065007	biological regulation	1.37E-26
BP	GO:0090304	nucleic acid metabolic process	1.49E-22
BP	GO:0006355	regulation of transcription, DNA-templated	3.24E-21
BP	GO:1903506	regulation of nucleic acid-templated transcription	3.24E-21
BP	GO:2001141	regulation of RNA biosynthetic process	3.24E-21
BP	GO:0051252	regulation of RNA metabolic process	3.31E-20
BP	GO:2000112	regulation of cellular macromolecule biosynthetic process	5.27E-20
BP	GO:0010556	regulation of macromolecule biosynthetic process	5.82E-20

BP	GO:0006310	DNA recombination	6.95E-20
BP	GO:0031326	regulation of cellular biosynthetic process	8.16E-20
BP	GO:0009889	regulation of biosynthetic process	8.16E-20
BP	GO:0019219	regulation of nucleobase-containing compound metabolic process	8.16E-20
BP	GO:0006139	nucleobase-containing compound metabolic process	2.13E-18
BP	GO:0006351	transcription, DNA-templated	4.15E-18
BP	GO:0097659	nucleic acid-templated transcription	4.15E-18
BP	GO:0032774	RNA biosynthetic process	4.84E-18
BP	GO:0010468	regulation of gene expression	8.21E-18
BP	GO:0006725	cellular aromatic compound metabolic process	1.22E-17
BP	GO:0046483	heterocycle metabolic process	1.22E-17
BP	GO:0007165	signal transduction	2.24E-17
BP	GO:0051171	regulation of nitrogen compound metabolic process	5.87E-17
BP	GO:1901360	organic cyclic compound metabolic process	6.16E-17
BP	GO:0080090	regulation of primary metabolic process	8.29E-17
BP	GO:0031323	regulation of cellular metabolic process	1.98E-16
BP	GO:0023052	signaling	2.01E-16
BP	GO:0007154	cell communication	5.1E-16
BP	GO:0060255	regulation of macromolecule metabolic process	9.43E-16
CC	GO:0016021	integral component of membrane	9.96E-16
CC	GO:0031224	intrinsic component of membrane	1.5E-15
BP	GO:0019222	regulation of metabolic process	4.66E-15
BP	GO:0034654	nucleobase-containing compound biosynthetic process	1.19E-14
BP	GO:0019438	aromatic compound biosynthetic process	3.55E-14
BP	GO:0051716	cellular response to stimulus	4.87E-14
BP	GO:0034641	cellular nitrogen compound metabolic process	4.93E-14
BP	GO:0018130	heterocycle biosynthetic process	5.72E-14
BP	GO:1901362	organic cyclic compound biosynthetic process	1.53E-13
CC	GO:0044425	membrane part	3.9E-12
BP	GO:0009987	cellular process	4.69E-12
BP	GO:0050896	response to stimulus	5.74E-11
BP	GO:0016070	RNA metabolic process	1.41E-10
BP	GO:0034645	cellular macromolecule biosynthetic process	4.19E-10
BP	GO:0009059	macromolecule biosynthetic process	4.78E-10
BP	GO:0044271	cellular nitrogen compound biosynthetic process	6.3E-10
BP	GO:0006259	DNA metabolic process	9.77E-10
BP	GO:0044260	cellular macromolecule metabolic process	7.26E-09
BP	GO:0010467	gene expression	6.92E-08
BP	GO:0044249	cellular biosynthetic process	1E-06
BP	GO:1901576	organic substance biosynthetic process	2.26E-06
BP	GO:0009058	biosynthetic process	3.97E-06
CC	GO:0016020	membrane	6.4E-06
MF	GO:0004984	olfactory receptor activity	1.02E-05

BP	GO:0043170	macromolecule metabolic process	4.6E-05
CC	GO:0005882	intermediate filament	0.000187
CC	GO:0045111	intermediate filament cytoskeleton	0.000187
MF	GO:0005200	structural constituent of cytoskeleton	0.000315
BP	GO:0008150	Biological process	0.000321
MF	GO:0016491	oxidoreductase activity	0.000667
BP	GO:0006807	nitrogen compound metabolic process	0.001245
BP	GO:0044237	cellular metabolic process	0.001337
MF	GO:0003677	DNA binding	0.004445
CC	GO:0099513	polymeric cytoskeletal fiber	0.004445
MF	GO:0003676	nucleic acid binding	0.006332
CC	GO:0099080	supramolecular complex	0.007018
CC	GO:0099081	supramolecular polymer	0.007018
CC	GO:0099512	supramolecular fiber	0.007018
BP	GO:0044238	primary metabolic process	0.010714
BP	GO:0071704	organic substance metabolic process	0.02769

Note: BP: biological process. MF: molecular function. CC: cellular component.

Supplementary Table 11. Results of the KEGG enrichment analysis of expanded gene families in the *C. versicolor* genome.

ID	Pathway	Adjusted P value
map05168	Herpes simplex infection	1.14E-85
map05202	Transcriptional misregulation in cancer	2.39E-82
map04020	Calcium signaling pathway	6.64E-81
map05169	Epstein-Barr virus infection	1.53E-78
map05414	Dilated cardiomyopathy (DCM)	2.03E-74
map05310	Asthma	5.89E-70
map05330	Allograft rejection	3.46E-66
map04672	Intestinal immune network for IgA production	2.9E-64
map05320	Autoimmune thyroid disease	3.09E-62
map04072	Phospholipase D signaling pathway	1.81E-60
map04145	Phagosome	5.03E-58
map05340	Primary immunodeficiency	3.04E-57
map05150	Staphylococcus aureus infection	1.13E-56
map05152	Tuberculosis	4.67E-55
map05416	Viral myocarditis	1.27E-53
map05323	Rheumatoid arthritis	1.6E-51
map05143	African trypanosomiasis	3.59E-51
map05322	Systemic lupus erythematosus	8.37E-51

map04064	NF-kappa B signaling pathway	1.2E-49
map04742	Taste transduction	3.06E-46
map04664	Fc epsilon RI signaling pathway	8.83E-44
map04662	B cell receptor signaling pathway	6.68E-42
map05130	Pathogenic Escherichia coli infection	1.7E-41
map04650	Natural killer cell mediated cytotoxicity	2.29E-40
map04640	Hematopoietic cell lineage	4.38E-40
map05140	Leishmaniasis	2.25E-39
map04666	Fc gamma R-mediated phagocytosis	3.07E-37
map05146	Amoebiasis	8.57E-36
map04151	PI3K-Akt signaling pathway	3.13E-26
map05332	Graft-versus-host disease	1.33E-08
map00830	Retinol metabolism	4.36E-08
map04940	Type I diabetes mellitus	1.85E-07
map05216	Thyroid cancer	4.02E-07
map05144	Malaria	9.43E-07
map05221	Acute myeloid leukemia	4.27E-06
map05321	Inflammatory bowel disease (IBD)	5.23E-06
map05213	Endometrial cancer	7.13E-06
map05217	Basal cell carcinoma	8.63E-06
map04612	Antigen processing and presentation	1.04E-05
map04658	Th1 and Th2 cell differentiation	8.97E-05
map04520	Adherens junction	9.47E-05
map04660	T cell receptor signaling pathway	0.000114
map05210	Colorectal cancer	0.00012
map05215	Prostate cancer	0.000147
map00140	Steroid hormone biosynthesis	0.00017
map04916	Melanogenesis	0.000187
map05412	Arrhythmogenic right ventricular cardiomyopathy (ARVC)	0.000196
map04659	Th17 cell differentiation	0.000216
map05142	Chagas disease (American trypanosomiasis)	0.000276
map00310	Lysine degradation	0.001405
map05162	Measles	0.001556

map04550	Signaling pathways regulating pluripotency of stem cells	0.002243
map04934	Cushing syndrome	0.004346
map05224	Breast cancer	0.005218
map04514	Cell adhesion molecules (CAMs)	0.006836
map04390	Hippo signaling pathway	0.007852
map05225	Hepatocellular carcinoma	0.011429
map04310	Wnt signaling pathway	0.011781
map05226	Gastric cancer	0.01214
